# Short term doxycycline treatment induces sustained improvement in myocardial infarction border zone contractility

**DOI:** 10.1371/journal.pone.0192720

**Published:** 2018-02-12

**Authors:** Kimberly Spaulding, Kiyoaki Takaba, Alexander Collins, Farshid Faraji, Guanying Wang, Esteban Aguayo, Liang Ge, David Saloner, Arthur W. Wallace, Anthony J. Baker, David H. Lovett, Mark B. Ratcliffe

**Affiliations:** 1 Veterans Affairs Medical Center, San Francisco, California, United States of America; 2 Department of Radiology, University of California, San Francisco, California, United States of America; 3 Department of Bioengineering, University of California, San Francisco, California, United States of America; 4 Department of Surgery, University of California, San Francisco, California, United States of America; 5 Department of Anesthesia, University of California, San Francisco, California, United States of America; 6 Department of Medicine, University of California, San Francisco, California, United States of America; Max Delbruck Centrum fur Molekulare Medizin Berlin Buch, GERMANY

## Abstract

Decreased contractility in the non-ischemic border zone surrounding a MI is in part due to degradation of cardiomyocyte sarcomeric components by intracellular matrix metalloproteinase-2 (MMP-2). We recently reported that MMP-2 levels were increased in the border zone after a MI and that treatment with doxycycline for two weeks after MI was associated with normalization of MMP-2 levels and improvement in *ex-vivo* contractile protein developed force in the myocardial border zone. The purpose of the current study was to determine if there is a sustained effect of short term treatment with doxycycline (Dox) on border zone function in a large animal model of antero-apical myocardial infarction (MI). Antero-apical MI was created in 14 sheep. Seven sheep received doxycycline 0.8 mg/kg/hr IV for two weeks. Cardiac MRI was performed two weeks before, and then two and six weeks after MI. Two sheep died prior to MRI at six weeks from surgical/anesthesia-related causes. The remaining 12 sheep completed the protocol. Doxycycline induced a sustained reduction in intracellular MMP-2 by Western blot (3649±643 MI+Dox vs 9236±114 MI relative intensity; p = 0.0009), an improvement in *ex-vivo* contractility (65.3±2.0 MI+Dox vs 39.7±0.8 MI mN/mm^2^; p<0.0001) and an increase in ventricular wall thickness at end-systole 1.0 cm from the infarct edge (12.4±0.6 MI+Dox vs 10.0±0.5 MI mm; p = 0.0095). Administration of doxycycline for a limited two week period is associated with a sustained improvement in *ex-vivo* contractility and an increase in wall thickness at end-systole in the border zone six weeks after MI. These findings were associated with a reduction in intracellular MMP-2 activity.

## Introduction

Segmental shortening in the border zone region adjacent to a myocardial infarction (MI) is depressed, even when the border zone has a normal blood supply [1]. While the decrease in border zone function was previously thought to be secondary to mechanical load [2], computational (finite element) modeling studies show that border zone contractility must be decreased by approximately 50% [3]. This finding has now been confirmed by studies in demembranated strips of myocardium obtained from the border zone after antero-apical MI in sheep [4].

The border zone in the current study is not the millimeter wide border zone comprised of interdigitating ischemic and normal myocardium described in the 1980s by Janse and others [5]. Rather, the border zone in the current study is a region with decreased contraction that is adjacent to the MI zone. It is larger than previously thought, extending as far as 3 cm from the edge of the infarct [6] and the region of dysfunction expands in size over time [1]. Progression of border zone size and dysfunction may contribute to post-MI ventricular remodeling and subsequent heart failure [1].

Matrix metalloproteinases (MMP) were initially defined by their involvement in remodeling of the extracellular matrix. However, a specific MMP, matrix metalloproteinase-2 (MMP-2) also acts within the cardiomyocyte. Two discrete isoforms of intracellular MMP-2 have been identified. The first consists of the full length MMP-2 (FL-MMP-2) isoform previously considered to only have a role in cardiac extracellular matrix remodeling. Several studies have shown that a significant fraction of synthesized FL-MMP-2 is retained in an enzymatically latent form within cardiomyocytes in direct association with sarcomeric components. Oxidative stress induced by ischemia/reperfusion injury can activate sarcomere-associated FL-MMP-2, an event that results in the cleavage of troponin I (TnI) [7], myosin light chain 1 (MLC-1) [8] and titin [9] with an associated reduction in contractile force. In addition, a second recently characterized isoform of MMP-2 is generated by oxidative stress-mediated activation of an alternate promoter located in the first intron of the MMP-2 gene. This event results in the synthesis of a N-**t**erminal truncated isoform of MMP-2 (NTT-MMP-2) that remains intracellular, is enzymatically active, and is physically associated with mitochondria [10]. Cardiac-specific transgenic expression of the NTT-MMP-2 isoform results in inflammation, cardiomyocyte necrosis and impaired contractility due to defects in calcium handling [11, 12].

Studies of MMP inhibitors on LV function and remodeling after MI in animals and humans have had mixed results. Doxycycline increases wall thickness in the MI border zone and decreases passive compliance after coronary artery occlusion MI in the rat [13]. Relatively selective inhibition of MMPs 2, 3 and 13 (PD166793) decreases infarct expansion after coronary artery occlusion MI in the pig [14]. Short-term doxycycline therapy reduced LV remodeling in patients with acute ST-elevation myocardial infarction (TIPTOP trial) [15]. In contrast, non-selective inhibition of MMPs 2, 3, 8, 9, 13 and 14 (PG116800) had no beneficial effect on post-infarction LV remodeling in humans (PREMIER trial) [16]. To date, no studies have looked at the effect of MMP inhibition on post-myocardial ventricular function in the normally perfused LV border zone.

Doxycycline (Dox) non-selectively inhibits MMPs by chelating the structural Zn^2+^ required for catalytic activity [17]. In addition, doxycycline can inhibit MMP transcription. Doxycycline also has free radical scavenger, anti-apoptotic [17] and immune modulatory effects [18].

We recently reported that treatment with doxycycline for two weeks after MI was associated with normalization of MMP-2 levels and improvement in *ex-vivo* contractile protein developed force in the myocardial border zone two weeks after infarction [4]. The purpose of the current study was therefore to determine if there is a sustained effect of short term treatment with doxycycline on border zone function in a large animal model of antero-apical MI.

## Methods

### Experimental animals

Sheep were acquired from a local ranch (Pozzi Ranch, Sebastapol, CA). A Q-fever titer was acquired prior to animal purchase and then twice during a three week quarantine at the animal facility. Only sheep with three negative titers were used.

Sheep were housed singly for 48 hours following each surgical procedure. They were then housed in pairs in runs (48 sq. feet) that had the floor covered with pine shavings and that were enriched with a salt block and a mirror. Housing was cleaned daily. Sheep were fed a prepared ruminant diet (Envigo, Huntington, Cambridgeshire, UK) and fresh alfalfa hay twice daily.

Sheep were checked daily and posture, temperature, respiratory rate, urine and fecal output and incision status were recorded. Sheep were weighed on arrival and then every two weeks.

#### Anesthesia and pain control

Anesthesia for MI, MRI and sacrifice/ tissue harvest procedures in chronic animals was similar. Briefly, anesthesia was induced with ketamine (20 mg/ kg intravenous) and maintained with isoflurane (2.2% inhaled). End-tidal CO_2_ was kept between 25 and 45 mmHg and an infusion of neosynephrine was titrated to keep peak LV pressure at 90±5 mm Hg during cardiac MRI and Swan Ganz catheter measurements.

Post-operative pain associated with the MI procedure was controlled with a transdermal fentanyl patch (1–2 ug/kg/hr continuous) started 24 hours prior to surgery and continued for 3 days after surgery. In addition, at the conclusion of MI and MRI procedures, the incision was infiltrated with a long acting local anesthetic (Bupivacaine 0.5% x 10 ml) and a non-steroidal anti-inflammatory (Banamine, 1.1 mg/kg) was given intra-muscularly once and as needed for post-operative pain.

#### Non-operated controls

Three adult unoperated sheep were sacrificed to obtain ‘non-operated’ control myocardial tissue. Those animals were given pentobarbital (150 mg/ kg IV) prior to rapid cardiectomy and tissue harvest as described below.

### Experimental protocol

Sheep were treated under a protocol approved by the San Francisco VA Institutional Animal Care and Use Committee (IACUC), in compliance with the “Guide for the Care and Use of Laboratory Animals” prepared by the Institute of Laboratory Animal Resources, National Research Council.

**Fig 1:**
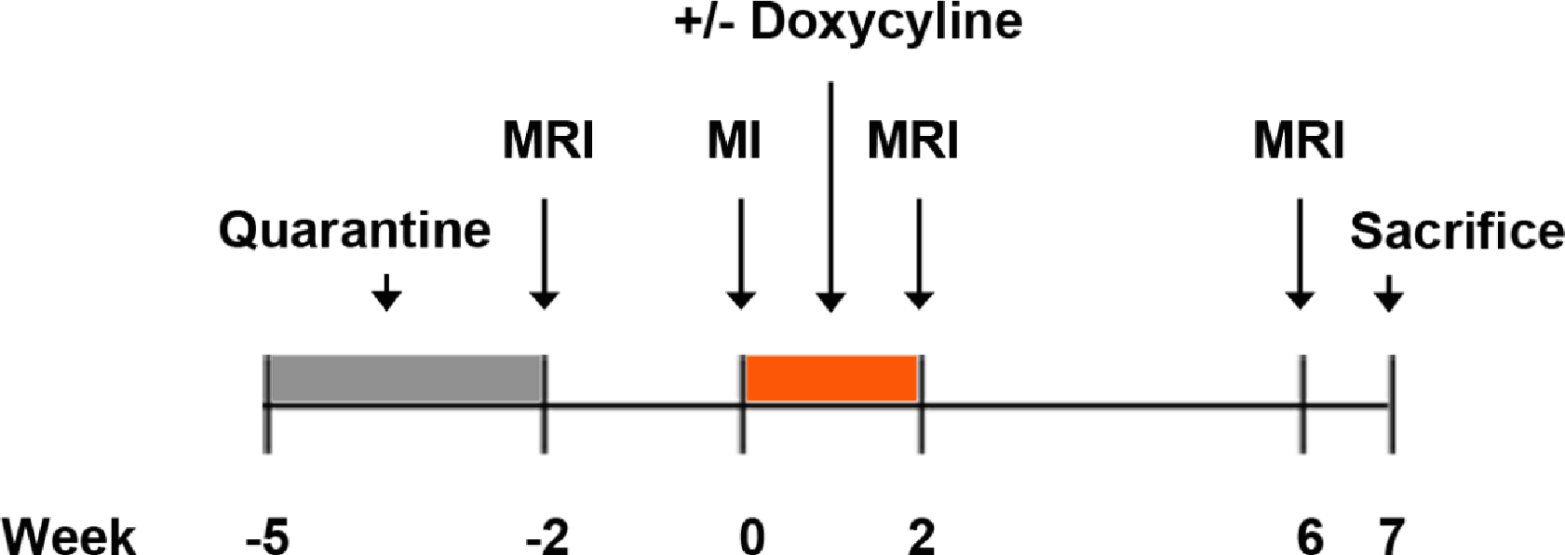
Experimental timeline.

Briefly, antero-apical MI was created in 14 sheep. Of those, seven sheep received doxycycline 0.8 mg/kg/hr IV for two weeks. Cardiac MRI was performed two weeks before, and then two and six weeks after MI (**Fig 1**).

#### Myocardial infarction

Fourteen adult sheep underwent antero-apical MI, as previously described [19]. The left anterior descending (LAD) and LAD diagonal branches were ligated at a point 40% of the distance from the LV apex to the base of the heart.

#### Doxycycline administration

Sheep designated for doxycycline treatment had an indwelling catheter (Groshong, Bard Medical, Covington, GA) implanted at the time of MI and advanced to the superior vena cava-right atrial junction. The catheter was tunneled to the animal’s back and connected to a continuous flow pump (Ambit, Sandy, UT). Sheep received doxycycline (0.8 mg/ kg/ hr or 19.2 mg/ kg/ day) IV continuously for 2 weeks. Doxycycline was started immediately prior to MI. The daily dose of doxycycline was identical to our previous study [4].

#### Cardiac magnetic resonance imaging (MRI)

**Fig 2:**
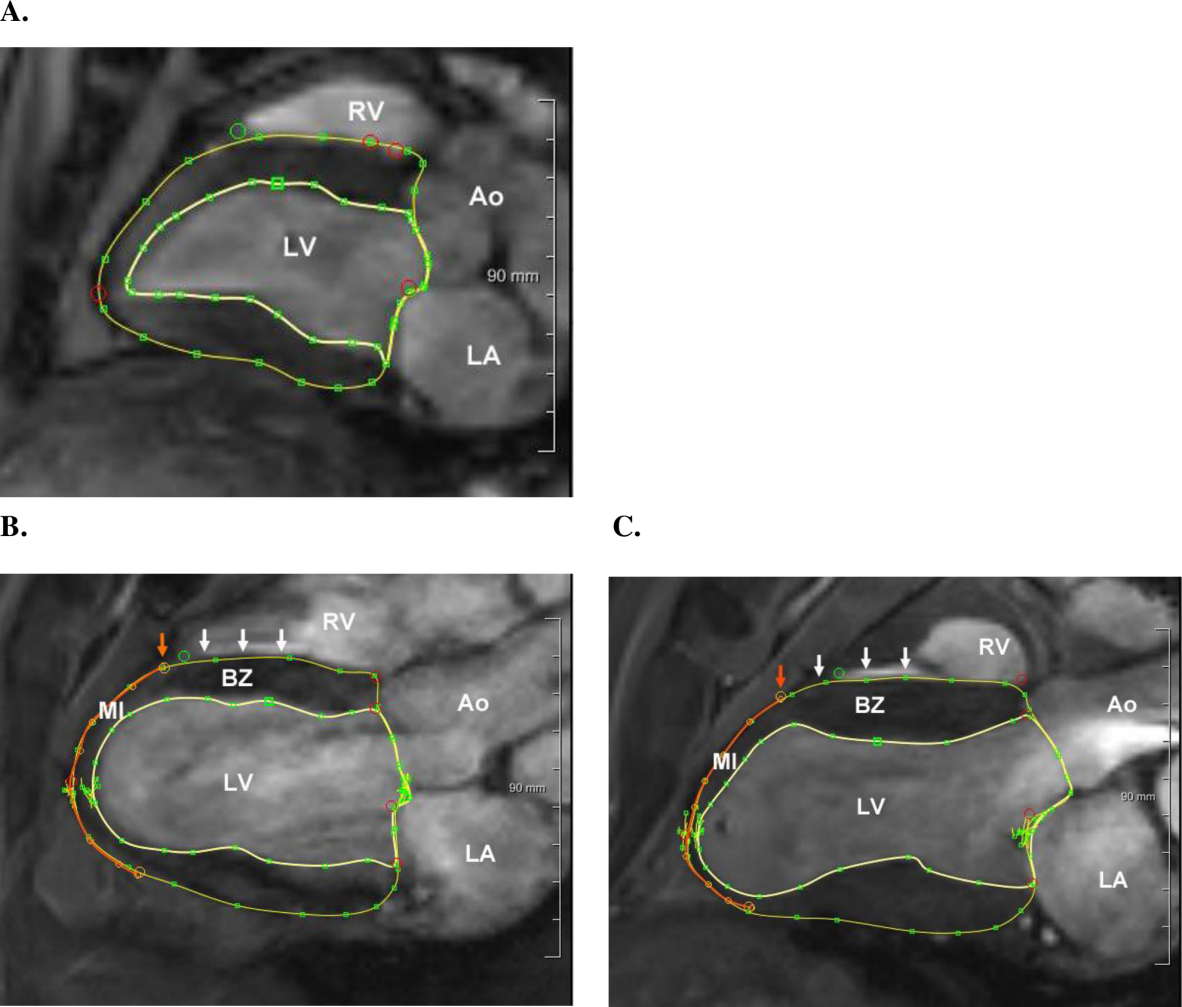
Representative MRI imaging data. Panel **A** is from control sheep two weeks prior to MI, panel **B** is from control animal six weeks after MI and panel **C** is from doxycycline animal six weeks after MI. All images are of the three-chamber long axis obtained at end systole. Epicardial and endocardial contour lines are in yellow. The red line is the manually determined MI perimeter. The red arrow marks the edge of the MI and white arrows mark the points of 1, 2, and 3 cm from the infarct edge.

Cardiac MRI was performed two weeks before, and then two and six weeks after MI, as described [20]. Briefly, six radial cine long axis images 30^o^ apart were obtained (**Fig 2**). ferumoxytol (0.125 ml/ kg IV over 1 hour; AMAG Pharmaceuticals, Waltham, MA) was given prior to MRI [21].

#### Swan Ganz catheter measurements

Following cardiac MRI, a Swan Ganz catheter was inserted and advanced to the pulmonary artery using fluoroscopic guidance. Cardiac output and pulmonary artery wedge pressure (PCWP) measurements were obtained.

#### Tissue harvest

Cardiac arrest was achieved by retrograde aortic infusion of 4°C hyperkalemic solution (Plegisol, Hospira Inc, Lake Forest, IL plus 10 mL/L 8.4% NaHCO_3_, pH 7.8). Tissue samples were taken from the MI, border zone and remote anterior wall for histology, muscle strip mechanics and biochemistry. The border zone tissue sample was obtained 0–1 cm from the infarct edge.

### MRI image analysis

**Fig 2** shows representative three-chamber long axis MRI images obtained at end-systole (ES) from control **(B)** and doxycycline **(C)** sheep 6 weeks after MI. LV endocardial and epicardial borders were manually contoured (MeVisLab, MeVis Medical Solutions, Bremen, Germany) and 3D triangular element surfaces created. Cavity and wall volumes of the LV at end diastole (ED) and ES were calculated (MeVisLab) [20]. LV volume measurements were indexed to body surface area [22] to the 1.5 power [23].

**Fig 3:**
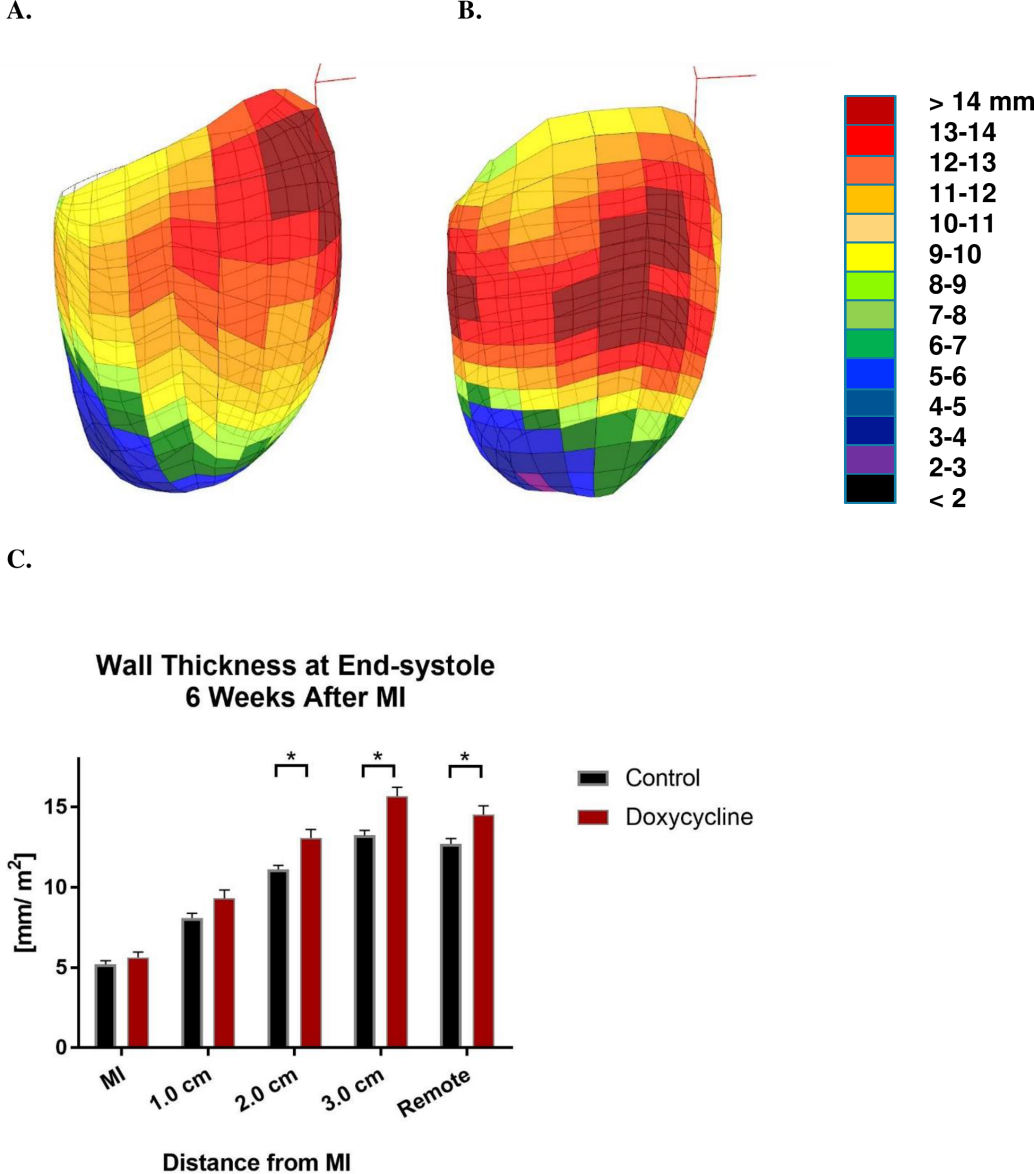
LV wall thickness at end-systole six weeks after MI. Panels **A** and **B** are color maps of LV wall thickness at end-systole six weeks after MI from representative MI control (**A**) and MI + Dox (**B**) animals. Maps are oriented with the anterior wall toward the viewer and colors range from black (< 2 mm) to brown (>14 mm). Panel **C** shows the effect of Doxycycline on wall thickness at end-systole in 1 cm increments from the MI edge. Note that data is from the entire LV. *p<0.05.

Points on the MI border were identified in each of the six long axis cine MRI images as the inflection between the region with contractile function and the constant thickness MI zone (**Fig 2B and 2C**). Epicardial and endocardial 3D spline contours were created from the MI border points and from points 3 cm toward the LV base on the LV epicardial and endocardial surfaces. A finite element mesh consisting of 1,728 hexahedral elements was projected to the epicardial and endocardial surfaces and designated elements were attached to the MI and border zone spline contours (TrueGrid, XYZ Corp, Livermore, Ca) (**Fig 3A** and **3B**). Wall thickness was determined by measuring the mesh thickness at the MI border and at centimeter increments along the LV border zone. Each measurement was repeated at each of the 24 elements around the circumference.

### *Ex-vivo* myofilament contractility

Thin slices of epicardial myocardium from border zone and remote myocardium were pinned onto a silicone substrate and chemically demembranated using a solution containing 1% Triton X-100 (Sigma-Aldrich, St Louis, MO) for 24 h at 4°C [4]. Demembranated slices were stored at -20°C (for up to several weeks before study) in a solution that was a 1:1 mixture of glycerol and relaxing solution containing (in mM) 20 EGTA, 7.05 MgCl2, 6.31 Na2ATP, 10 creatine phosphate, and 80 N,N-bis(2-hydroxyethyl)2-aminoethane sulfonic acid, with pH adjusted to 7.1 with KOH and ionic strength adjusted to 200 mM with KCl. Relaxing solution also contained 1% (vol/vol) Protease Inhibitor Cocktail P-8340 and 10 IU/ml creatine kinase (Sigma).

Developed force of demembranated myocardial samples was measured as described [4]. Visually discrete cardiac muscle bundles (diameter 100 μm—150 μm) running in the plane of the slice were dissected free. Muscle bundles were cut to a length of 1.5 mm and attached with aluminum T-clips to a force transducer (Permeabilized Fiber Test System, model 1400A, Aurora Scientific, Aurora, ON, Canada) and bathed in relaxing solution. Muscle sarcomeres were observed using a 40X objective, and sarcomere lengths were assessed using a video-based system (model 900B, Aurora Scientific). Muscle length was adjusted to set the muscle sarcomere length to 2.1 μm. Demembranated preparations were briefly transitioned to preactivating solution in which Ca^2+^ buffering was reduced by replacing 19.5 mM EGTA with hexamethylenediamine-*N*,*N*,*NV*,*NV-*tetraacetate (Honeywell Fluka, Seelze, Germany). Muscles were then transitioned to an activating solution containing 20 mM Ca^2+^-EGTA. Maximum developed force was assessed from the contraction force in activating solution minus the passive force measured in relaxing solution, normalized to the muscle cross-sectional area (mN/mm^2^). Data were pooled from two to four muscle preparations obtained per region per heart.

### MMP-2 immunohistochemistry

Immunohistochemistry of FL-MMP-2 and NTT-MMP-2 isoforms was performed as described [24]. In brief, to detect the FL-MMP-2 isoform we used an antibody to the N-terminal prodomain of MMP-2 (AB54401, Abcam, Cambridge, MA). For immunostaining of the NTT-MMP-2 isoform, the sections were incubated overnight at 4° C with an affinity-purified goat IgG (5μg IgG/ml PBS/BSA) targeting the S1’ substrate binding sequence (P^347^YTYTKNFRLSQDD^361^) located adjacent to the catalytic site [24].

### MMP-2 western blot

Western blotting was performed on protein isolated from fresh-frozen border zone tissue using antibodies to FL-MMP-2 and NTT-MMP-2 that were previously described [24]. Relative abundance of the MMP-2 protein was normalized to GAPDH.

### MMP-2 qPCR

Paraffin-embedded sections (15 μm) of anterior border zone tissue were deparaffinized and RNA was extracted using an RNeasy FFPE kit (Qiagen, Hilden, Germany). RNA was quantified and normalized to synthesize cDNA using the Maxima First Strand cDNA Synthesis kit for RT-qPCR, with dsDNase (Thermo Fisher Scientific, Waltham, MA. The relative expression of FL-MMP-2 and NTT-MMP-2 transcripts between border zone tissue from MI, MI + Dox and non-operated controls were determined using a LightCycler 480 SYBR Green I Master kit (Roche Applied Science, Penzberg, Germany). Samples were plated in triplicate in 384 well PCR plates (Thermo Fisher Scientific). Primers were designed to assay for the sheep FL-MMP-2 transcript (*F*: *5’–AGATGCAGAAGTTCTTGGGT– 3’,* *R*: *5’–CTCTGGTCCAGTTCACCTGT– 3’*) and the NTT-MMP-2 transcript (*F*: *5’–GTGTGCTTGCAAGAAGTGGG– 3’,* *R*: *5’–GTCCAGTTCACCTGTCTGGG– 3’)*. Amplification reactions were performed with 40 cycles (95°C for 15 seconds; 56°C for 30 seconds; and 72°C for 1 minute) and normalized to the ribosomal protein L19 (RPL19) transcript (*F*: *5’–AGCCTGTGACTGTCCATTCC– 3’*, *R*: *5’–ACGTTACCTTCTCGGGCATT– 3’)*. ^25^ Comparative results are expressed using the 2 ΔΔ Ct method [25].

### Fluorescent imaging of reactive oxygen species (ROS)

Samples from the anterior border zone and remote myocardium were snap-frozen in liquid nitrogen cooled isopentane and stored at -80°C prior to analysis. Intracellular superoxide was measured with the fluorescent probe dihydroethidium (DHE; Molecular Probe, Eugene, OR) [26]. Hydroxide ion was measured using dichlorofluorescein (DCF; Molecular Probe) [26] in 8.0μm thick frozen sections as described. Images of ROS stained sections were analyzed using ImageJ (NIH, Bethesda, MD). Specifically, the respective ROS stain was the percent area that was positive with a green filter.

### Histology

Samples from the border zone and remote myocardium were fixed in phosphate-buffered 4% paraformaldehyde (Thermo Fisher Scientific) at 4°C overnight, followed by storage in 70% ethanol at 4°C. Paraffin-embedded sections (5 μm) were stained with hematoxylin and eosin.

### Statistical analysis

All values are expressed as mean ± standard error of the mean. The significance level was set at p<0.05.

A multivariate mixed effect analysis (Proc Mixed, SAS version 9.2, SAS Institute Inc., Cary, NC) was performed. Individual sheep were included as a random effect [27]. The Bonferroni method was used to correct for multiple comparisons.

## Results

Average initial animal weights (MI: 62.0±1.9 and MI+Dox: 56.2±2.9 Kg) and weight gain (MI: 4.9 and MI+Dox: 7.3 Kg) in the two groups were similar (**Table 1)**.

**Table 1:** Cardiac MRI measurements.

	2 weeks prior to MI (n = 14)	2 weeks after MI (n = 7)	2 weeks after MI +Dox (n = 7)	6 weeks after MI (n = 7)	6 weeks after MI +Dox (n = 5)
**Weight [Kg]**	59.1±2.1	61.8±2.4	57.9±2.8	66.9±2.6	63.5±4.4
**Long axis at ED [mm]**	85.5±1.9	91.9±7.6	91.1±9.7	91.3±1.9	97.2±4.0*
**Short axis at ED [mm]**	38.3±1.5	42.2±7.1	44.2±2.6	45.4±4.6	49.9±2.7*
**Sphericity at ED**	0.45±0.02	0.470±0.112	0.490±0.070	0.497 ± 0.048	0.517±0.034
**Apical Conicity at ES**	0.700±0.04	0.510±0.071*	0.510 ± 0.035*	0.546±0.024*	0.555±0.037*
**MI Perimeter at ED [mm]**	NA	7.8±0.7	7.9±0.69	7.4±0.4	7.9±0.7
**MI Area at ES [cm**^**2**^**]**	NA	NA	NA	4.96±0.38	5.31±0.56
**LV Volume Index at ED [ml/ m**^**2**^**]**	108.3±5.2	136.7±6.2*	142.1±11.0*	145.0±10.5*	151.1±17.0*
**LV Volume Index at ES [ml/ m**^**2**^**]**	63.2±3.4	91.4±6.3*	103.1±7.8*	103.3±7.6*	108.6±13.3*
**LV Wall Volume at ED [ml]**	127.9±5.5	117.3±7.5	130.0±8.7	146.0±5.4	143.4±5.8
**Ejection Fraction [%]**	41.4±2.3	33.5±2.6	27.2±3.4	28.2±4.3*	28.5±4.2*

Values are expressed as mean ± standard error of the mean. LV volumes are indexed to body surface area to the 1.5 power. ^23^ED-end-diastole; ES-end-systole.

* = p < 0.05 with respect to 2 weeks prior to MI.

Two animals died immediately prior to MRI study six weeks after MI from anesthesia related causes. The remaining 12 animals completed the protocol.

### Effect on regional LV function

The effect of Doxycycline on regional LV wall thickness at end-systole six weeks after MI is seen in **Fig 3.** Color maps of LV wall thickness from representative MI control (**A**) and MI + Dox (**B**) animals show a generalized increase in wall thickness at ES across the border zone and remote LV myocardium. Panel **C** shows the effect of Doxycycline has a statistically significant effect on wall thickness at end-systole beginning 2 cm above the MI border and extending into the remote myocardium. Doxycycline had no statistically significant effect on wall thickness at end-diastole (Data not shown).

### Effect on global LV function and shape

**Table 1** shows measurements obtained from cardiac MRI. Briefly, the long and short axes, and LV volume at ED trended larger with doxycycline, but the changes were not significant. Both LV sphericity and conicity were not different after doxycycline treatment.

MI perimeter and MI area trended higher with Doxycycline. As a consequence, and in spite of the increase in wall thickness at ES in the border zone and remote myocardium, LV volume at ES was not significantly different.

**Table 2** shows Swan Ganz catheter measurements, including stroke volume and PCWP. Stroke volume trended higher and PCWP trended lower with doxycycline treatment; however, the changes were not significant. Multivariate analysis of a composite created by adding stroke volume and PCWP values normalized with pre-MI mean and standard deviation trended toward a modest doxycycline effect (p = 0.075).

**Table 2:** Swan Ganz catheter measurements.

	2 weeks prior to MI (n = 14)	2 weeks after MI (n = 7)	2 weeks after MI +Dox (n = 7)	6 weeks after MI (n = 7)	6 weeks after MI +Dox (n = 5)
**Heart Rate [bpm]**	83.6±6.4	89.6±10.0	74.3±3.4	81.6±7.3	81.4±7.4
**Cardiac Output [L/min]**	5.6±0.4	5.3±0.6	4.4±0.7	4.8±0.4	5.3±0.5
**Stroke Volume [ml]**	68.4±5.0	60.4±5.7	59.2±9.0	60.9±5.0	69.0±11.2
**PCWP [mm Hg]**	5.8±0.6	13.3±2.3	10.9±1.5*	11.6±1.7	7.8±1.9

Values are expressed as mean ± standard deviation. PCWP-pulmonary capillary wedge pressure.

*p < 0.05 with respect to 2 weeks prior to MI.

### *Ex-vivo* myofilament contractility

**Fig 4:**
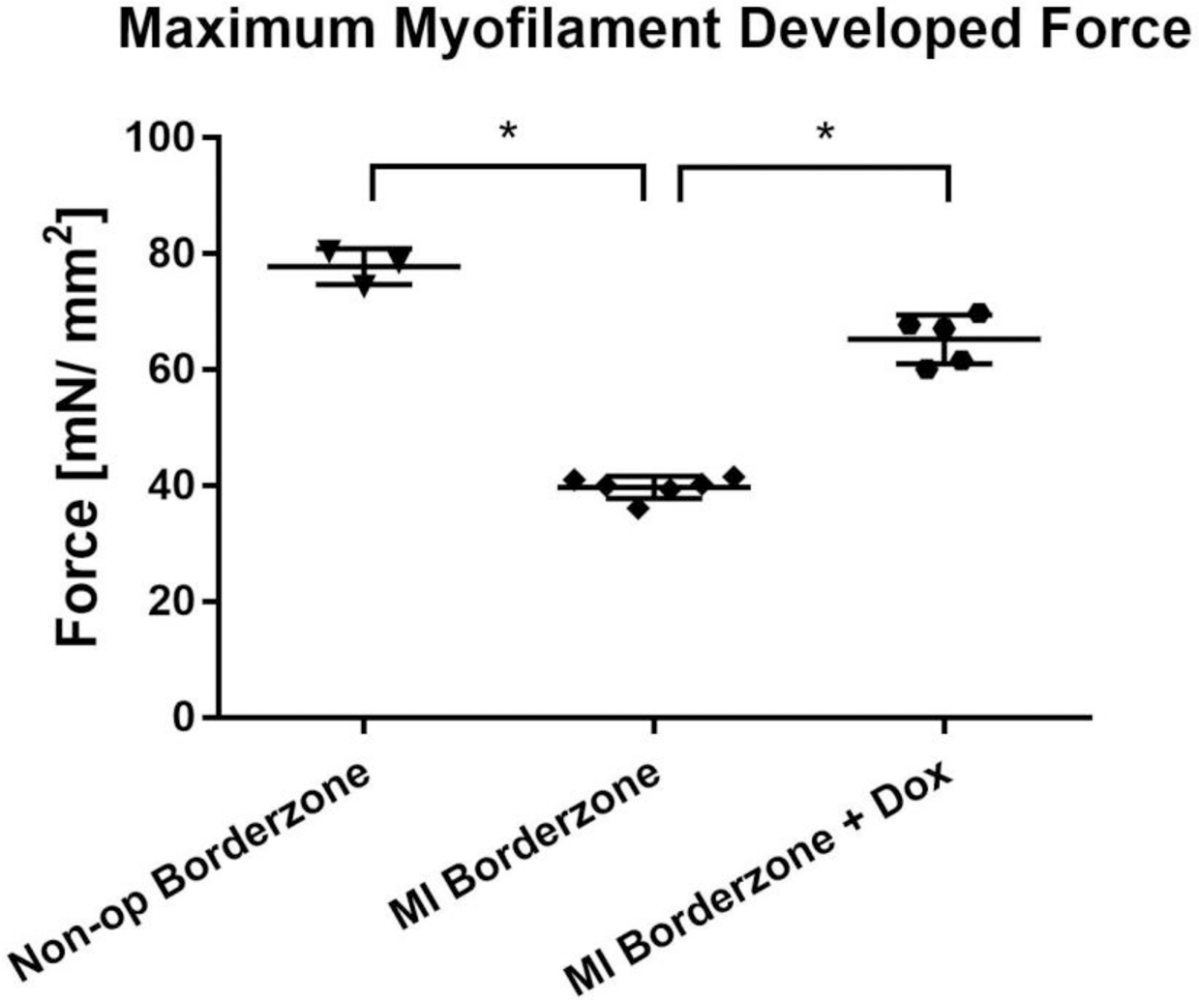
Effect of doxycycline on maximum myofilament developed force six weeks after MI. The statistical comparison is with MI border zone control. * = p < 0.0001 with respect to MI border zone.

**Fig 4** shows the effect of doxycycline on maximum myofilament developed force assessed using demembranated cardiac muscle preparations from the infarct border zone six weeks after MI. Maximum developed force in the infarct border zone was 52.7% (p<0.001) of remote myocardium and 59.9% (p<0.001) in myocardium from non-operated sheep. Developed force in border zone myocardium of doxycycline treated sheep was 65% (p<0.0001) higher than the border zone after MI and not different from remote myocardium or border zone of non-operated controls. Passive force was unchanged by MI or doxycycline (not shown).

### Intracellular MMP-2

**Fig 5:**
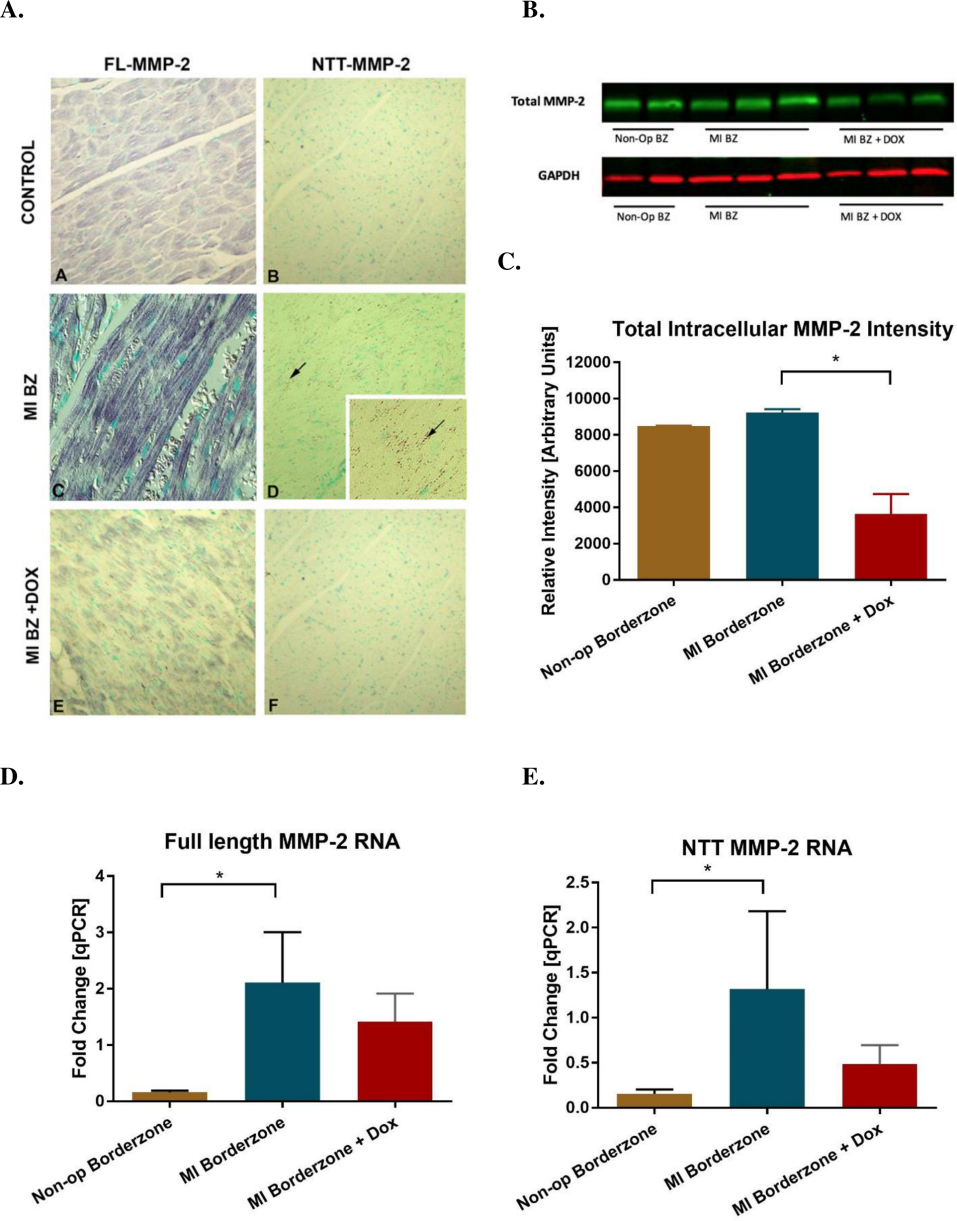
Effect of doxycycline on MMP-2 in the infarct border zone six weeks after MI. Panel **A** shows the effect on MMP-2 using immunohistochemistry and panels **B** and **C** show western blot results. Panel **A** subpanels **C** and **D** were done using Nomarski optics. FL-MMP-2 staining is sarcomeric while the NTT-MMP-2 staining shows punctate, linear arrays of antibody staining between sarcomeres (black arrows). *p< 0.05. The effect of doxycycline on full length (**D**) and truncated (**E**) MMP-2 RNA. *p<0.05 for combined FL-MMP-2 and NTT-MMP-2 RNA effect. n = 4 for MI control and MI + Dox groups, n = 2 for non-operated control.

**Fig 5A** shows the effects of doxycycline on immunohistochemical staining for the FL-MMP-2 and NTT-MMP-2 isoforms in the infarct border zone 6 weeks after MI. Cardiomyocyte staining for the FL-MMP-2 isoform in the controls shows strong immuno-histochemical reaction product decorating sarcomeres, while NTT-MMP-2 staining was present in particulate, linear arrays characteristic of a mitochondrial localization, as previously reported [11]. Doxycycline treatment significantly reduced expression of both MMP-2 isoforms as determined by immunohistochemistry.

**Fig 5B** shows the effect of doxycycline on MMP-2 protein level by Western blot using a MMP-2 antibody that recognizes both the FL-MMP-2 and NTT-MMP-2 isoforms. Given the limits on molecular mass resolution of SDS-PAGE gels, it is not possible to clearly distinguish between the 68 kDa FL-MMP-2 and the 65 kDa NTT-MMP-2 protein isoforms and the results are expressed in terms of total intracellular MMP-2 protein content. Compared to the non-operated controls, total MMP-2 protein was not changed in the infarct border zone, but was decreased by 60.5% (p = 0.0009) in the infarct border zone in the doxycycline treated group as compared to MI control.

### MMP-2 qPCR

**Fig 5D** and **5E** show an increase in expression of the FL-MMP-2 and NTT-MMP-2 transcripts in the MI border zone after MI (combined effect: p = 0.0184) and a trend toward reduction with doxycycline (combined effect: p = 0.0646). Both were relative to the normalizing L19 ribosomal protein gene.

### ROS

**Fig 6:**
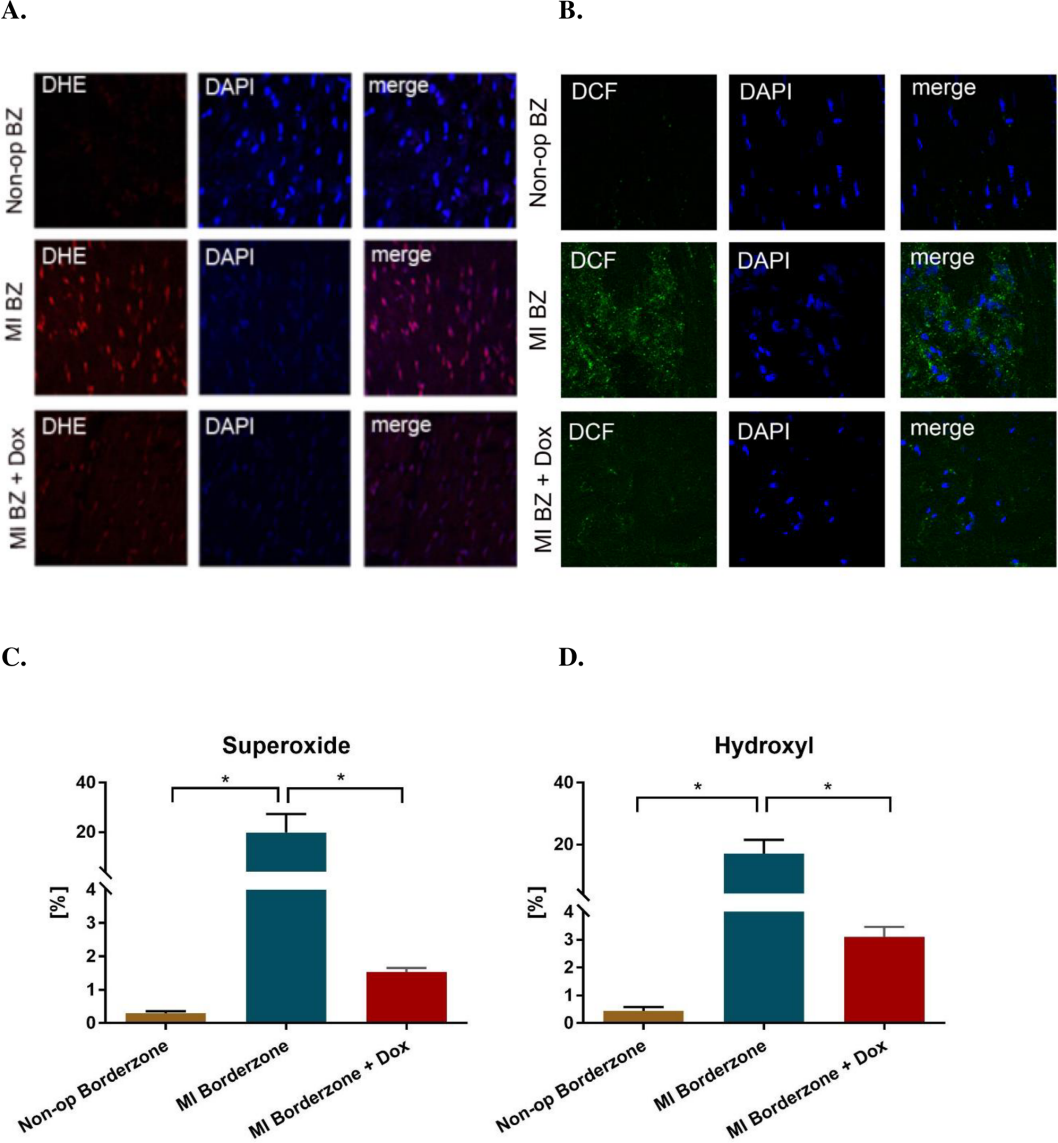
Effect of doxycycline on reactive oxygen species in the infarct border zone six weeks after MI. Panel **A** shows the effect on superoxide and panel **B** on hydroxide. Superoxide was measured dihydroethidium (DHE) and hydroxide was measured with dichlorfluorescein (DCF). DAPI = 4',6-diamidino-2-phenylindole. Panels **C** and **D** show the % area of superoxide (DHE) and hydrogen peroxide (DCF) staining respectively. n = 3 for MI control and MI + Dox groups, n = 2 for non-operated control.

**Fig 6** shows the effect of doxycycline on superoxide and hydroxide production in the border zone six weeks after MI. There was minimal staining for superoxide in the non-op control myocardium. After MI, there was significant superoxide activity co-localized to the myocyte nucleus and hydroxide activity localized in a punctate pattern consistent with mitochondrial localization (Panels **A** and **B**; MI BZ Merge). Treatment with doxycycline was associated with a significant reduction in superoxide and hydroxide in both the remote and border zone myocardium (Panels **C** and **D**).

## Discussion

The principal finding of the study is that administration of doxycycline for a limited two week period following acute MI is associated with a sustained improvement in *ex-vivo* contractility and an increase in wall thickness at end-systole in the border zone six weeks after MI. The effect is associated with a reduction in intracellular expression of both the FL-MMP-2 and NTT-MMP-2 isoforms.

### Proposed mechanism

Our proposed mechanism is that increased intracellular ROS activates existing intracellular FL-MMP-2 [28] and induces transcription of NTT-MMP-2 [10]. Activated FL-MMP-2 causes lysis of sarcomeric proteins leading to decreased myofilament force development and reduced LV function. Oxidative stress not only actives FL-MMP-2 but also stimulates transcription through an AP-1 enhancer element located in the promoter of the MMP-2 gene [29, 30]. Similarly, oxidative stress induces NTT-MMP-2 transcription through a NFκB enhancer element located near to the proximal promoter in the first intron of the MMP-2 gene. NTT-MMP-2 subsequently impairs mitochondrial bioenergetics and induces a primary innate immune response associated with cardiomyocyte death and decreased contractile force due to defects in calcium handling. The source of intracellular ROS in the infarct border zone is likely a result of NTT-MMP-2 induced mitochondrial dysfunction or alternatively mechano-transduction of increased mechanical stress via NADPH Oxidase (NOX-2) [31].

### Doxycycline effect

During the period of drug administration, doxycycline directly inhibits FL-MMP-2 and NTT- MMP-2 activity via chelation of structural Zn^2+^ required for catalytic activity [17]. The decrease in NTT MMP-2 activity is associated with a reduction in mitochondrial damage which in turn prevents the amplification of intracellular ROS production. Doxycycline may also act as a direct free radical scavenger [17]. Last, the doxycycline mediated reduction in intracellular ROS generation prevents transcriptional amplification of both MMP-2 isoforms. These proposed actions are consistent with the finding that MMP-2 mRNA and protein levels and intracellular ROS were significantly decreased in doxycycline-treated animals.

The sustained improvement in border zone contractility after a limited two week period of doxycycline administration requires comment. Given that the half-life of doxycycline is approximately 15–18 hours, it is very unlikely that the sustained beneficial effect of a two week course of doxycycline treatment is a consequence of persistence of doxycycline in the infarct border zone at four weeks following discontinuation of administration [32].

A corollary is that the stimulus for intracellular ROS production by either mitochondrial dysfunction or mechano-transduction of high mechanical stress must be short lived or else ROS and MMP-2 activity would recur in the absence of doxycycline. Cytokines, such as TNF-α [33], and monocytes are known to rapidly increase in remote myocardium after MI in the mouse. Monocyte numbers peak on day 10 following MI and then decline precipitously [34]. Infiltration of inflammatory cells in the border zone has not been measured but is assumed to be similar.

Along those lines, it is interesting that the doxycycline effect is sustained given that mechanical stress and strain in the border zone remain abnormal after the acute post- MI period [1, 35] and NOX-2 should therefore continue to generate the ROS and downstream MMP-2 effects described above. Possibly, NOX-2-based mechano-transduction ceases late after MI due to accommodation to mechanical stress.

### Contractile protein dysfunction in the infarct border zone

Shimkunas and colleagues recently showed that maximal developed force generated by demembranated myocardium dissected from the anterior border zone of sheep two weeks after MI was decreased by 34% [4]. First, comparison with the results of this study where maximum myofilament developed force from the border zone six weeks after MI was decreased by 46% suggests that contractile protein dysfunction is a progressive time dependent phenomena. Further, Shimkunas found that dysfunction of myofilaments from the infarct border zone involved impaired cross bridge formation and decreased myosin light chain-1 phosphorylation, possibly due to the action of FL-MMP-2 [4]. Although not measured in this study, the same mechanism may be operative six weeks after MI.

### Border zone dysfunction *in vivo*

In addition to contractile protein dysfunction per se [4], defects in calcium handling machinery [36], and energy available to support contraction [37] are present *in vivo* in the region adjacent to a myocardial infarction. Lovett and colleagues recently studied cardiomyocyte contractility in intact cells taken from NTT-MMP-2 transgenic mice [11]. Lovett found that myofilament force generation was unchanged but intact cell force generation and the amplitude of Ca^2+^ transients were decreased by 50%. These findings are consistent with predominant localization of the NTT-MMP-2 in sub-sarcolemmal mitochondria [11] While we did not measure Ca^2+^ transients or high energy phosphate levels in this study, the increase in NTT-MMP-2 in the border zone in the current study suggests that NTT-MMP-2 upregulation contributes to border zone contractile dysfunction *in vivo*.

### Effect on global LV anatomy and function

These experiments were done in part to determine the effect of improved border zone function after MI on global LV pump function. To that end, we previously simulated the effect of an improvement in border zone contractility on end-systolic elastance and Starling’s law using finite element models based on MRI images of five sheep after antero-apical MI [38]. With a 50% increase in border zone contractility, those simulations predicted a decrease in LV volume at end-systole of 5.8 ml at 90 mm Hg afterload and an increase in stroke volume of 4.6 ml at LV end-diastolic pressure of 10 mm Hg [38]. In retrospect, those predictions were partially accurate as stroke volume increased by 6.6 ml with doxycycline (**Table 2**). However, the increase in stroke volume was associated with a trend toward increased LV volume. The reasons for this are unclear but infarct wound healing and subsequent infarct expansion are likely affected by doxycycline.

### Limitations

There are several limitations in the present study. First, specific downstream targets of FL-MMP2 including MLC-1, TnI, Titin and targets of NTT-MMP-2 including Ca^2+^ handling and mitochondrial function were not measured. In addition, implantation of an MRI compatible marker at the infarct edge at the time of MI would have allowed more accurate measurement of the effect of doxycycline on infarct expansion.

### Conclusion and future directions

The principal finding of the study is that administration of doxycycline for two weeks is associated with a sustained improvement in *ex-vivo* contractility and increase in wall thickness at end-systole in the border zone six weeks after MI. The effect is likely mediated by a reduction in intracellular MMP-2 activity.

Future studies using more selective MMP inhibition and ROS scavenging agents are warranted. Direct border zone therapy could also be part of multimodality treatment strategies that include therapies designed to limit infarct expansion including passive constraint of the infarct.
